# Supplementary material for: Transcriptome Analysis of Response to Zika Virus Infection in Two Aedes albopictus Strains with Different Vector Competence
Source: Int J Mol Sci. 2023 Feb 21;24(5):4257. doi: 10.3390/ijms24054257 (PMC10002152; doi:10.3390/ijms24054257)
Supplement: Supplementary file 1 [file ijms-24-04257-s001.zip › Figure S1-S4.pdf]

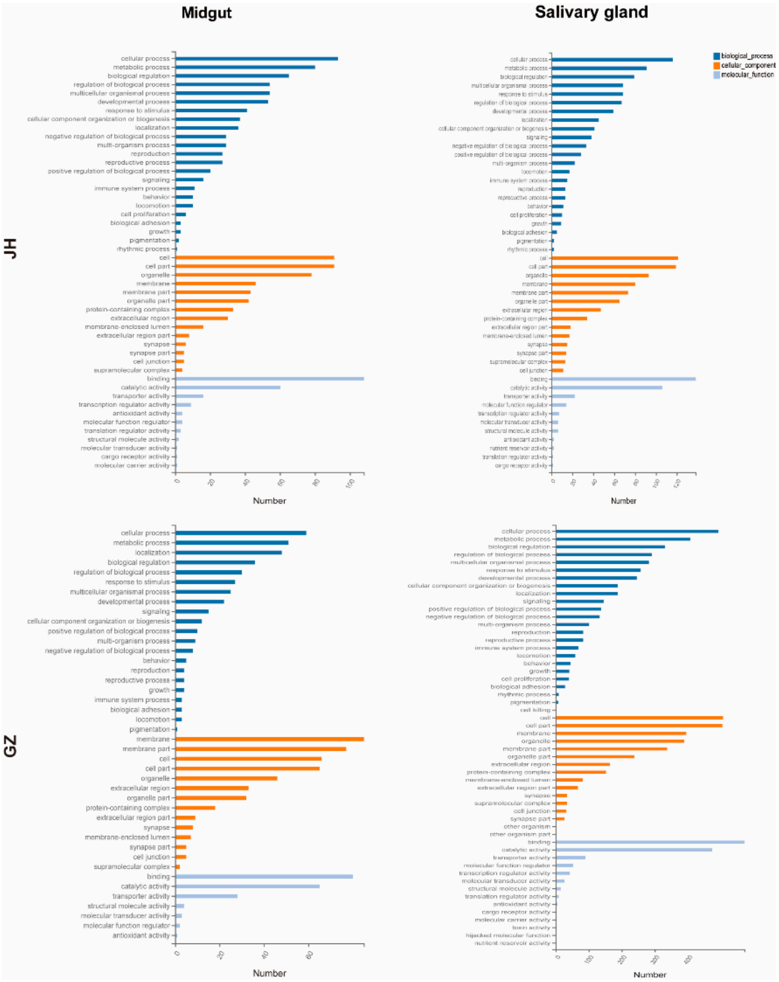

Figure S1. GO analysis of DEGs. Blue, orange, and light blue bars indicate biological processes (BP), cellular components (CC) and molecular functions (MF), respectively.

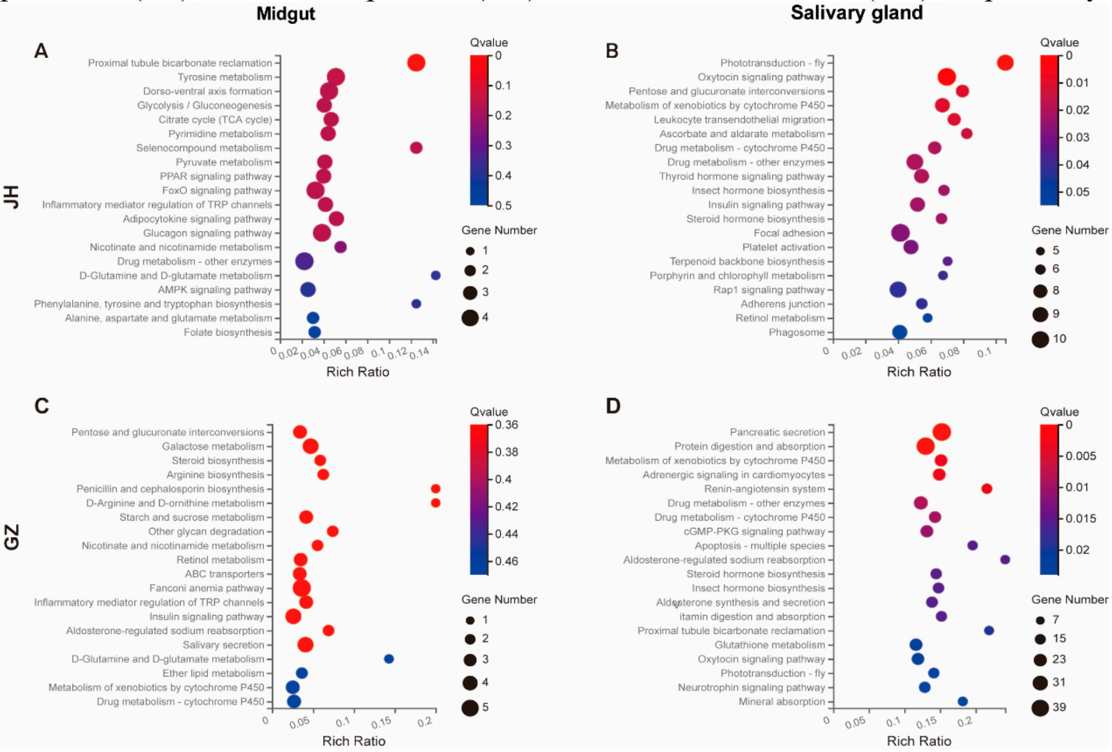

Figure S2. KEGG enrichment analysis of DEGs. The Y axis was the item of KEGG

pathway and the X axis was the enrichment ratio. Rich Ratio = the number of genes annotated to an item in the DEGs of this library / the total number of genes annotated to the same item in the species. The color represents the Qvalue of the enrichment and the size represents the number of genes.

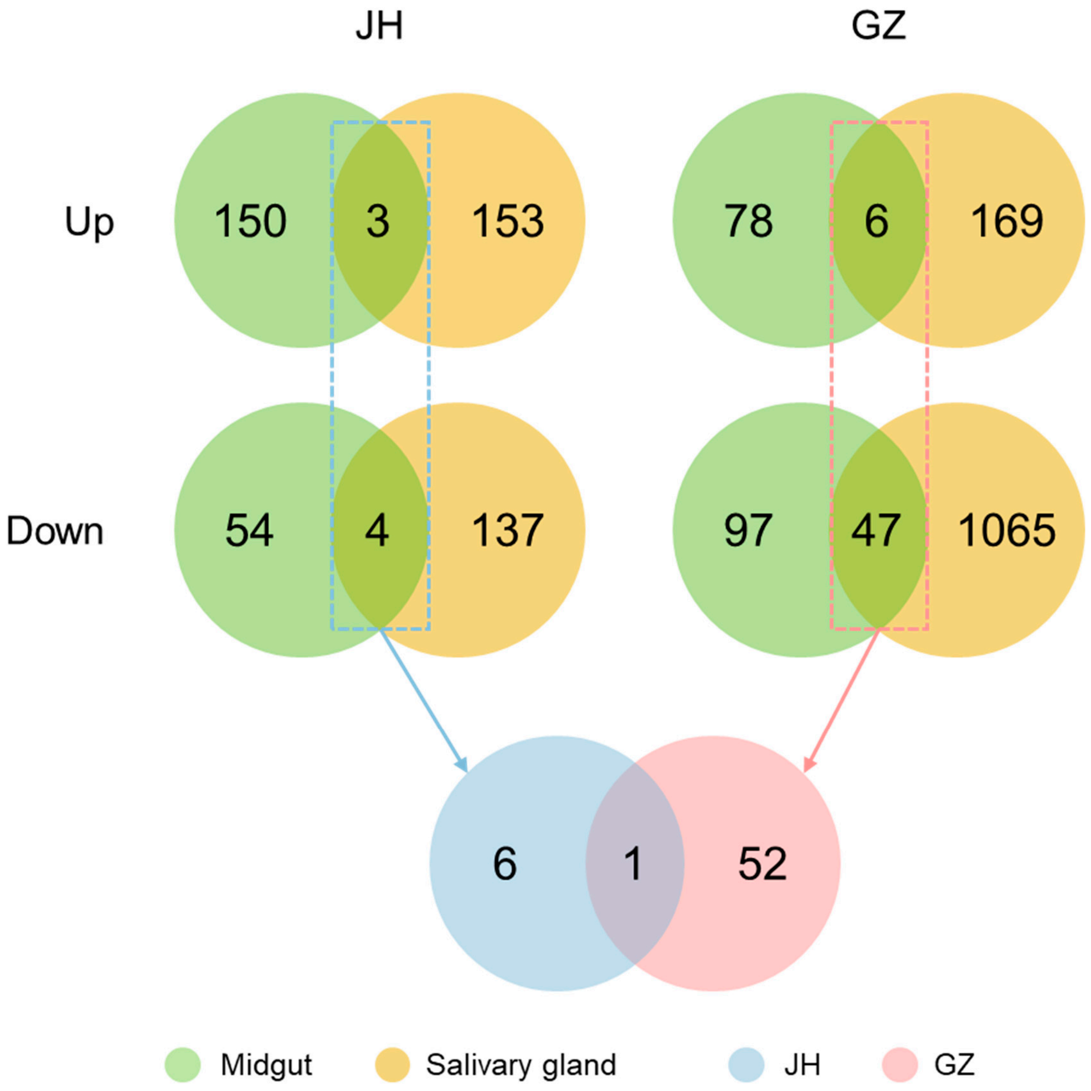

Figure S3. Venn diagram representing the number of DEGs in the different groups.

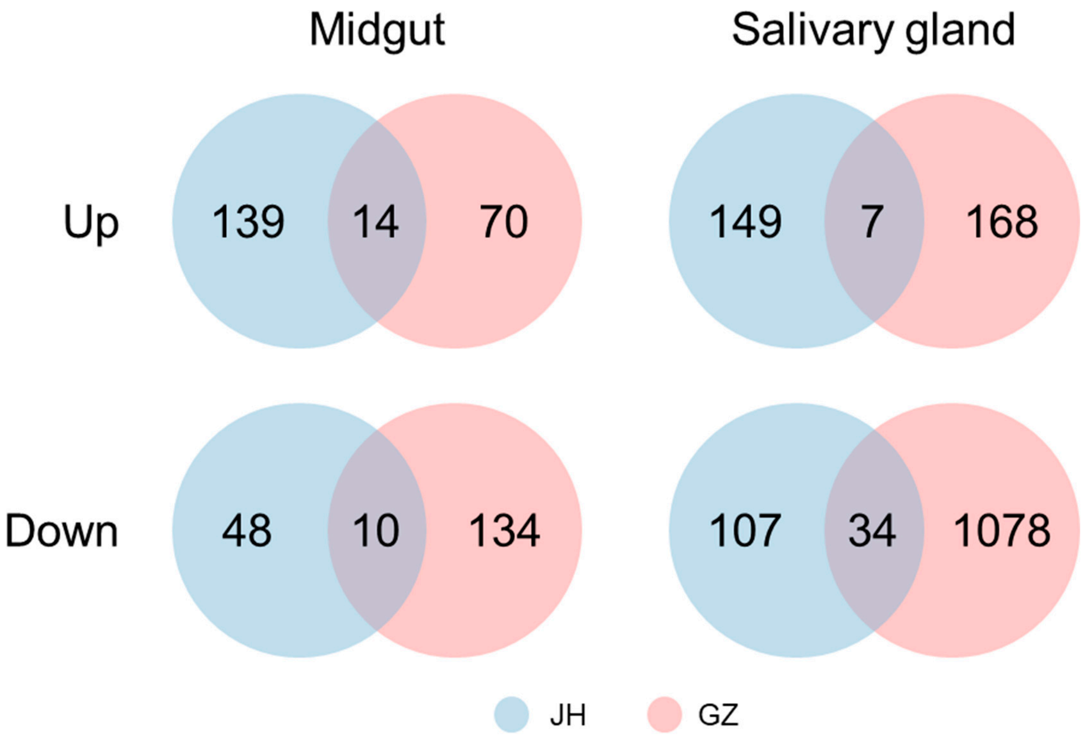

Figure S4. Venn diagram representing the number of DEGs in two *Ae. albopictus* JH and GZ strains after ZIKV infection.
