# Supplementary material for: Transcriptome Analysis of Response to Zika Virus Infection in Two Aedes albopictus Strains with Different Vector Competence
Source: Int J Mol Sci. 2023 Feb 21;24(5):4257. doi: 10.3390/ijms24054257 (PMC10002152; doi:10.3390/ijms24054257)
Supplement: Supplementary file 1 [file ijms-24-04257-s001.zip › Table S1ú║ Primer sequences of selected mRNA transcripts and the reference gene.pdf]

Table S1. Primer sequences of selected mRNA transcripts and the reference gene.

Gene	Orientation	Sequence	Length (bp)
LOC109426042	F	CTGGGGATGTGACTCGTTGT	162
	R	AGGTCCGAGAAGTTGAGCAC	
LOC109418949	F	TGCCACACTACAAACGGGAA	113
	R	TGGTCGGTAGTAGGAGTCGG	
LOC115257102	F	TGTGTGAGTGTGTCGCACCA	125
	R	ACGAGGGTAGAGACGGGAA	
LOC109399074	F	TGTTTCCTGGCTATGTGCCT	90
	R	AGAATTGGCAACAGGACGGG	
LOC109411713	F	CGCGCTTCGGGATTATGTTG	80
	R	GATCTGTCGCTGCTCTGGAA	
LOC109414797	F	CTGGTGTCAGCCTACGTCC	143
	R	CCATTCGACAGGAGCGGTG	
LOC109415384	F	GAATCCTCAGACGCACCACA	137
	R	GATGTGCTTACCTGCTCCGT	
LOC109419968	F	GATACCAACTGGACGGGACC	106
	R	CGGTCTCGGGTGTACTGAAG	
LOC109431492	F	CTGATACTGTTGAGCCGCCTACT	145
	R	GTTTCACTGGTTGGGTCTTGG	
LOC109405426	F	TTGCTGGATGTGCTTCGGTAT	148
(CYP304a1)	R	CTTGCCGCTGGAATCGCTC	
actin	F	TCCCACACAGTCCCCATCTAC	121
	R	ACGAGTAGCCACGTTCAGTCAG	
