# Supplementary material for: Transcriptome Analysis of Response to Zika Virus Infection in Two Aedes albopictus Strains with Different Vector Competence
Source: Int J Mol Sci. 2023 Feb 21;24(5):4257. doi: 10.3390/ijms24054257 (PMC10002152; doi:10.3390/ijms24054257)
Supplement: Supplementary file 1 [file ijms-24-04257-s001.zip › Table S2ú║ siRNA sequences of CYP304a1 and GFP.pdf]

Table S2. siRNA sequences of CYP304a1 and GFP.

Gene	SS Sequence	AS Sequence
CYP304a1	GACUGGACAUAGUCAACAACC	UUGUUGACUAUGUCCAGUCCG
GFP	GGUGUUCAAUGCUUCUCAAGA	UUGAGAAGCAUUGAACACCAU
