# Supplementary material for: Transcriptome Analysis of Response to Zika Virus Infection in Two Aedes albopictus Strains with Different Vector Competence
Source: Int J Mol Sci. 2023 Feb 21;24(5):4257. doi: 10.3390/ijms24054257 (PMC10002152; doi:10.3390/ijms24054257)
Supplement: Supplementary file 1 [file ijms-24-04257-s001.zip › Table S3ú║The sample size of siRNA microinjection, ZIKV infection and ZIKV detection.pdf]

Table S3. The sample size of siRNA microinjection, ZIKV infection and ZIKV detection.

Strains	JH		GZ	
siRNA	GFP	CYP304a1	GFP	CYP304a1
Number of mosquitoes injected with siRNA	125	142	121	150
Number of dead post siRNA	70	92	30	70
Number of mosquitoes injected with ZIKV	43	41	74	73
Number of dead post ZIKV injection	26	18	38	34
Number of mosquitoes tested on 1dpi	7	7	10	12
Number of mosquitoes tested on 3dpi	10	16	11	15
